# Silica–Polymer Heterogeneous Hybrid Integrated Mach–Zehnder Interferometer Optical Waveguide Temperature Sensor

**DOI:** 10.3390/polym16162297

**Published:** 2024-08-14

**Authors:** Zhanyu Gao, Yuhang Du, Qizheng Zhang, Yinxiang Qin, Jiongwen Fang, Yunji Yi

**Affiliations:** 1 College of Integrated Circuits and Optoelectronic Chips, Shenzhen Technology University, Shenzhen 518118, China; 2210412050@email.szu.edu.cn (Z.G.); 202100302042@stumail.sztu.edu.cn (Y.D.); 202201201059@stumail.sztu.edu.cn (Q.Z.); 202201202022@stumail.sztu.edu.cn (Y.Q.); 2 College of New Materials and New Energies, Shenzhen Technology University, Shenzhen 518118, China; 202100301015@stumail.sztu.edu.cn

**Keywords:** temperature sensor, polymer optical waveguide, silica optical waveguide, hybrid integration

## Abstract

In this paper, a temperature sensor based on a polymer–silica heterogeneous integrated Mach–Zehnder interferometer (MZI) structure is proposed. The MZI structure consists of a polymer waveguide arm and a doped silica waveguide arm. Due to the opposite thermal optical coefficients of polymers and silica, the hybrid integrated MZI structure enhances the temperature sensing characteristics. The direct coupling method and side coupling method are introduced to reduce the coupling loss of the device. The simulation results show that the side coupling structure has lower coupling loss and greater manufacturing tolerance compared to the direct coupling structure. The side coupling loss for PMMA material-based devices, NOA material-based devices, and SU-8 material-based devices is 0.104 dB, 0.294 dB, and 0.618 dB, respectively. The sensitivity (S) values of the three hybrid devices are −6.85 nm/K, −6.48 nm/K, and −2.30 nm/K, which are an order of magnitude higher than those of an all-polymer waveguide temperature sensor. We calculated the temperature responsivity (R_T)_ (FSR→∞) of the three devices as 13.16 × 10^−5^ K, 32.20 × 10^−5^ K, and 20.20 × 10^−5^ K, suggesting that high thermo-optic coefficient polymer materials and the hybrid integration method have a promising application in the field of on-chip temperature sensing.

## 1. Introduction

Sensors are the core devices of the Internet of Things (IOT). With the development of new energy vehicles, wearable electronic devices, and intelligent robots, high-security temperature detection is indispensable in predicting potential risks to batteries and monitoring circuit temperature distribution. Compared to electrical temperature sensors, optical temperature sensors have a variety of advantages, such as immunity to electromagnetic interference, high security, and multiplexed detection. Therefore, optical temperature sensors have become an important solution to the problem of temperature detection.

Optical temperature sensors can be divided into fiber optic temperature sensors and planar optical waveguide temperature sensors. Among them, fiber optic sensors can be used for signal transmission and measurement of physical quantities. Their intuitive response to changes in physical parameters makes them suitable for monitoring tunnels, bridges, mines, pipelines, and other environments requiring long-distance distributed sensing. Compared with fiber optic sensors, planar optical waveguide sensors have the advantages of high integration, diversified choices of device structures and materials, and compatibility with semiconductor processes. Optical waveguide temperature sensors can be categorized based on their structure as follows: Mach–Zehnder interferometer (MZI) [1], microring resonators (MRR) [2], and waveguide Bragg gratings (WBG) [3]. WBGs are made by etching the grating on the waveguide structure. When the external temperature changes, it leads to changes in the refractive index of the waveguide material and the period of the grating, which makes the detection spectrum drift, and ultimately achieves the function of temperature detection through the monitoring of the spectral drift. In 2016, Benéitez et al. [3] designed a hybrid organic/inorganic waveguide Bragg grating temperature sensor, which was tested to reach a sensitivity of −249 pm/°C, a result that was 25 times better than that of a conventional silica fiber optic sensor. However, the grating size is not easy to control and requires a spectrometer. The MRR is based on the change in the surrounding temperature, which makes the ring cavity length and effective refractive index change, and then leads to the drift of the resonance wavelength, and ultimately achieves the function of detecting the temperature. In 2016, Hyun-Tae Kim et al. [2] designed a dual-cascaded ring resonator, which had a temperature sensitivity of 293.9 pm/°C. However, it requires high machining accuracy, and the cost of spectral analysis is high. Compared to microrings and gratings, the MZI waveguide sensor has distinctive advantages, such as high sensitivity [4], a simple design [5], and an easy fabrication process. The working principle is as follows: the structural design makes the two interfering arms of the MZI have different optical range differences. When the ambient temperature changes, the optical signals show different phase changes when transmitted through the two interfering arms of the MZI, and finally, the coherent interference outputs the optical signals at the output port, which change with the temperature. Researchers can further improve the sensing performance of polymer devices by improving and optimizing the length, width, and material of the two arms of the MZI structure. In 2018, Donghai Niu et al. [6] designed an MZI polymer waveguide sensor by varying the length of the two arms of the waveguide, and the results showed that the device with a length difference of 92 μm had a maximum sensitivity in the temperature interval ranging from 25 °C to 75 °C. The sensitivity could reach −431 pm/°C. In 2019, Donghai Niu et al. [7] also enhanced the sensitivity of the device by varying the width of the two arms of the waveguide. The sensitivity of the waveguide temperature sensor was measured to be 30.8 nm/°C when the width difference was 6.5 μm.

Currently, the main materials for the production of integrated optical devices include Silicon on Insulator (SOI), silica (SiO_2_), silicon nitride (SiN), polymer materials, and so on. Different materials have different properties. SOI is one of the most commonly used optical materials due to the large refractive index difference between the core and cladding layers, which is conducive to the limitation of the optical field and thus the reduction in the device size, as well as the fact that its preparation process is compatible with the traditional semiconductor process, Complementary Metal Oxide Semiconductor (CMOS). In 2021, Zuoqin Ding [8] fabricated MZI optical waveguide temperature sensors on an SOI platform with a sensitivity of up to 445 pm/°C. However, SOI material’s characteristics make it difficult to prepare devices that require high thermal stability, and its complex and high-cost preparation process is not conducive to widespread adoption. Silica waveguides and single-mode fibers exhibit a good fitting relationship, with very low transmission loss between them (−0.02 dB/cm) [9]. However, the small refractive index difference between the waveguide core layer and the cladding layer often leads to a larger device size, which is not conducive to integration. Although silicon nitride has a lower loss, the complexity of the preparation process and high cost are not conducive to large-scale application. Compared with inorganic materials, polymer materials exhibit superior thermo-optic effects [10]. These properties enable the fabrication of thermo-optic devices that effectively reduce power consumption, as well as temperature sensors that enhance sensitivity. Furthermore, polymer materials have advantages, such as low cost [11], ease of modification, simple processing [12], and good compatibility [3]. Therefore, optical waveguide devices based on polymers are an important component of photonic integrated devices [13]. In 2017, Yu Liu et al. [14] designed an all-polymer optical waveguide sensor based on polymer PMMA as the substrate and SU-8 as the core layer, and achieved average and linear sensitivities of 768.1 dB/RIU and 548.95 dB/RIU. In 2019, Donghai Niu et al. [15] proposed a material-asymmetric temperature sensor based on the Mach–Zehnder interferometer (MZI) structure. They demonstrated that the sensor exhibits temperature sensitivity by using materials with different thermo-optic coefficients but similar refractive indices in the two interfering arms of the MZI. Sensitivity was assessed by monitoring the output light intensity of the device, which simplifies the testing and operational processes and facilitates practical applications. The temperature sensitivity of the device was measured to be −1.685 °C^−1^.

In order to realize the complementary advantages of several materials, hybrid integration can be a solution [16]. In 2016, Xiaowei Guan et al. [17] designed an MZI-based temperature sensor with one of the arms using SU-8 cladding. SU-8 has a negative thermo-optic coefficient (−1.21 × 10^−4^ K^−1^). Thus, when the temperature varies, the phase change of the two arms is reversed, and its sensitivity is increased by up to 172 pm/°C. In 2022, D. A. Payne et al. [18] fabricated a heterogeneously integrated MZI temperature sensor with two arms composed of silicon and silicon nitride waveguides, whose thermo-optical coefficients differed significantly by an order of magnitude. The sensor demonstrated a sensitivity of 324 pm/K, a threefold improvement compared to an MZI utilizing only silicon waveguides on the same device.

In this paper, we report a temperature sensor utilizing an MZI with silica and polymer waveguides in each arm. Due to the positive thermo-optic coefficient of silica, while the polymer exhibits a negative thermo-optic coefficient, the temperature-induced phase changes in the two interfering arms of the MZI occur oppositely. Compared to an MZI composed of identical materials in both arms, the output optical signal of the MZI presented in this paper exhibits more pronounced variations with temperature changes, resulting in higher device sensitivity. Furthermore, the refractive index of silica is comparable to that of optical fiber materials, leading to reduced coupling loss. According to the coupling modes of the devices, we designed two structures based on direct coupling and one structure based on side coupling and employed three polymer materials to develop seven different sensors. We optimized the coupling structure and analyzed its sensitivity and coupling loss. The sensors exhibit high manufacturing tolerance, low loss, and exceptional sensitivity and have great potential for application in the field of temperature sensing.

## 2. Design of the Device Structure

**Figure 1:**
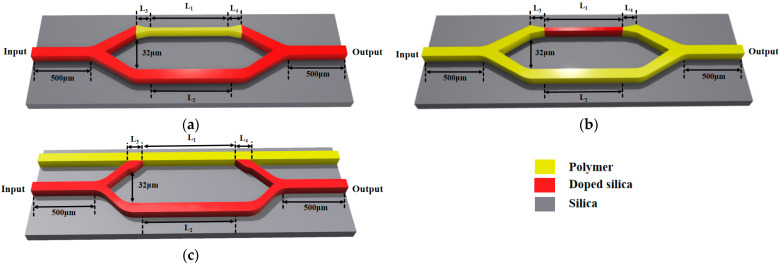
(**a**) The schematic of structure 1; (**b**) the schematic of the structure 2; (**c**) schematic of structure 3.

In this paper, three different device structures are designed. Structures 1 and 2 both utilize a direct coupling method, while structure 3 utilizes a side coupling method. Structure 1, shown in Figure 1a, consists of silica input and output single-mode waveguides, a 3 dB silica Y-branch beam splitter and combiner, a silica reference arm, and a polymer sensing arm. Parameters W_1_ and W_2_ represent the widths of the input and output tapers, while L_3_ and L_4_ are the lengths of these tapers. Structure 2, shown in Figure 1b, includes polymer input and output single-mode waveguides, a 3 dB silica Y-branch beam splitter and combiner, a polymer reference arm, and a silica sensing arm. It has the same parameters W_1_, W_2_, L_3_, and L_4_ as structure 1. Structure 3, shown in Figure 1c, utilizes doped silica material for a straight waveguide as the reference arm, while the remaining components, including the input and output single-mode waveguides, the 3 dB Y-branch beam splitter and combiner, and the polymer sensing arm, are made of polymer material. Parameters L_3_ and L_4_ in this structure are the same as structure 1 and structure 2, which are the lengths of the input and output tapers.

**Figure 2:**
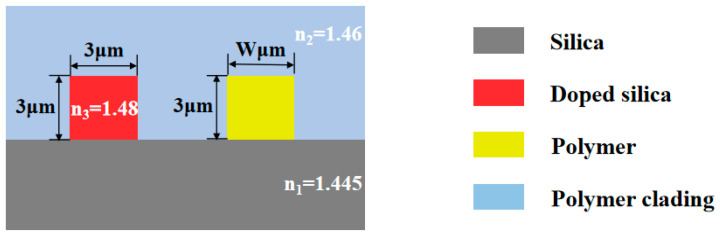
The cross-sectional diagram of the sensor.

The cross-sectional schematic of the device is shown in Figure 2. The device is built on a silica substrate, which has a refractive index of n_1_ = 1.445 at a wavelength of 1550 nm. This material also exhibits a thermal conductivity of 1.4 W/mK and a thermo-optic coefficient of 1 × 10^−5^ K^−1^. The upper cladding layer is made of a polymer material with a refractive index of n_2_ = 1.46. The inorganic part of the waveguide core layer is doped SiO_2_, with a refractive index of n_3_ = 1.48 at a wavelength of 1550 nm, and a thermo-optic coefficient of 1 × 10^−5^ K^−1^. For the polymer component of the core layer, three distinct polymer materials were selected: PMMA, NOA, and SU-8. In the manufacture of optical devices, SU-8, NOA, and PMMA materials each exhibit distinct advantages. SU-8 is characterized by its high optical transparency, excellent resolution, precise structure fabrication capabilities, mechanical strength, thick-film formation ability, low thermal expansion coefficient, and resistance to chemicals and UV radiation. These properties make it particularly suitable for applications demanding high precision, stability, and durability. NOA is characterized by its high optical transparency, superior refractive index matching, strong adhesion, rapid curing properties, low moisture absorption, and minimal optical loss, which are advantageous for applications requiring high precision and stability, especially in micro- and nanoscale processing. PMMA is characterized by its high transparency, excellent optical uniformity, ease of processing, cost-effectiveness, mechanical strength, light weight, coating capability, and environmental stability, making it well suited for a variety of optical applications. According to Equations (1), Equation (2) and Equation (3), the refractive indices of these polymers at 1550 nm are calculated to be 1.495 for PMMA, 1.538 for NOA, and 1.571 for SU-8, with thermo-optic coefficients of −1.2 × 10^−4^ K^−1^, −3 × 10^−4^ K^−1^, and −1.8 × 10^−4^ K^−1^, respectively.

(1) $$n=1.491+0.003427\lambda^{-2}+0.0001819\lambda^{-4},$$

(2) $$n=1.5375+0.00829045\lambda^{-2}-0.000211046\lambda^{-4},$$

(3) $$n=1.566+0.00796\lambda^{-2}+0.00014\lambda^{-4},$$

**Figure 3:**
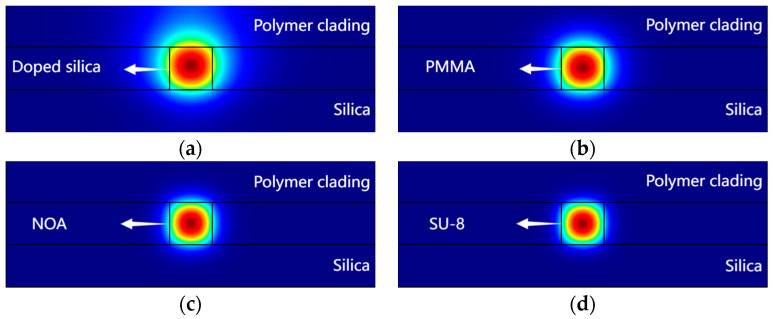
The optical field distribution of the (**a**) doped silica arm; (**b**) PMMA arm; (**c**) NOA arm; (**d**) SU-8 arm.

Considering that larger dimensions of the waveguide bring more modes and smaller dimensions increase coupling loss between the device and the fiber, the core layer dimensions of the waveguide were selected as 3 μm × 3 μm. Under these dimensions, the optical field distribution of the cross-sections of the waveguide can be obtained through the finite element method (FEM). Since the shape of the light field depends on the refractive index difference, the most intense part of the internal light intensity is typically circular, while the outer boundary is influenced by the refractive index difference between the core and the cladding, as shown in Figure 3. The mode power of all four waveguides in this study is well limited in the core region; a larger difference between the refractive index of the core and the cladding results in less light leakage and a light field that more closely matches the geometry of the waveguide.

## 3. Optimization of the Coupling Structure

Silica waveguides and SU-8 waveguides exhibit distinct propagation constants and coupling coefficients. This difference can result in optical field leakage during coupling due to mode mismatch, leading to losses. Under the condition that the mode power of both the polymer and silica waveguides was effectively confined to the core region, we optimized the coupling tapers between these waveguides to reduce coupling losses.

### 3.1. Direct Coupling Structure

**Figure 4:**
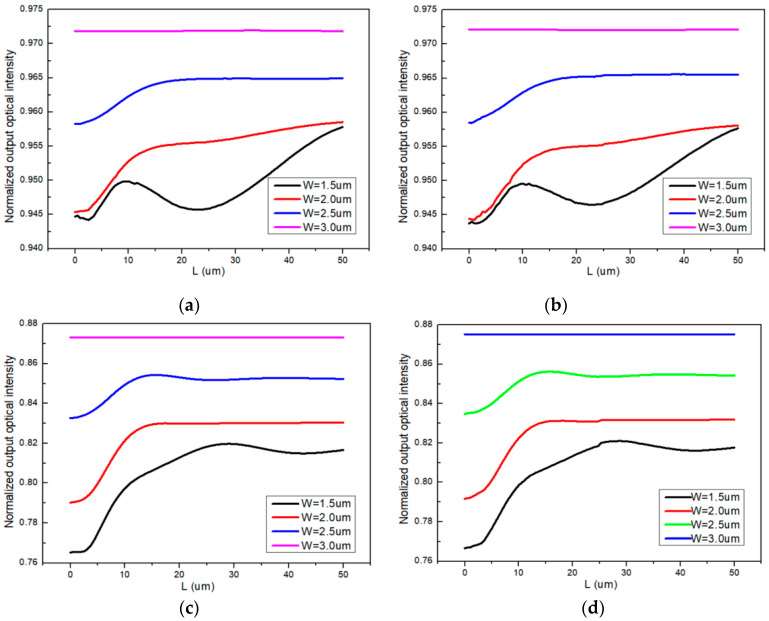
Normalized output light intensity with different taper widths and lengths (**a**) input taper based on PMMA material; (**b**) output taper based on PMMA material; (**c**) input taper based on NOA material; (**d**) output taper based on NOA material.

We utilized the beam propagation method (BPM) to optimize the length and width of the input and output tapers to reduce coupling loss. Due to the mode mismatch, coupling between SU-8 and silica waveguides results in significant loss; therefore, PMMA and NOA materials were used in structure 1 and structure 2, respectively. As shown in Figure 4a,b, for PMMA-based waveguides, with input and output taper widths W_1_ = W_2_ = 3 μm (length being variable), the coupling loss was measured at 0.371 dB and 0.123 dB. Similarly, for NOA-based waveguides, with W_1_ = W_2_ = 3 μm (length being variable), the coupling loss was measured at 0.589 dB and 0.579 dB, as shown in Figure 4c,d.

According to the simulation results in Figure 4, we designed four sensors. Structure 1 was utilized for sensor 1 and sensor 2, with PMMA and NOA chosen as the polymer materials for sensor 1 and sensor 2, respectively. Structure 2 was utilized for sensors 3 and 4, with PMMA and NOA also chosen as the polymer materials for sensor 3 and sensor 4, respectively. To reduce coupling loss in these four sensors, the lengths of their reference and sensing arms were L_1_ = L_2_ = 5000 μm, the widths of the input and output tapers were W_1_ = W_2_ = 3 μm, and the lengths of the input and output tapers were L_3_ = L_4_ = 3 μm.

**Figure 5:**
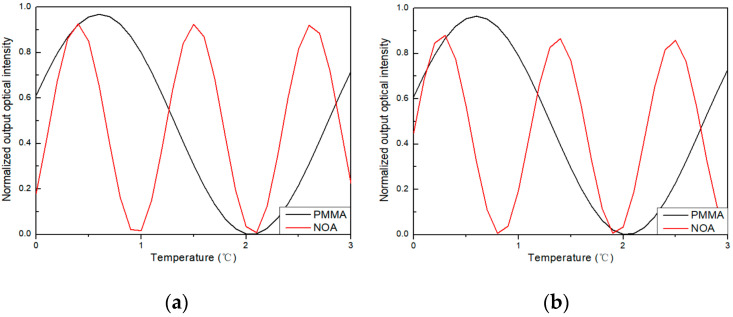
The relationship between the normalized output optical intensity with temperature at wavelengths of 1550 nm. (**a**) Sensor 1 and sensor 2; (**b**) sensor 3 and sensor 4.

Next, the temperature sensing performance of the four sensors was analyzed by the BPM. The relationship between normalized output light intensity and temperature at a wavelength of 1550 nm is shown in Figure 5. In Figure 5a, the black line represents the simulation data of sensor 1, and the red line represents sensor 2. The temperatures required for achieving π-phase modulation were 1.4 °C and 0.5 °C, with a loss of 0.138 dB and 0.339 dB, respectively. Similarly, in Figure 5b, the black line represents sensor 3, while the red line represents sensor 4. The temperatures required for achieving π-phase modulation were 1.4 °C and 0.5 °C, with a loss of 0.155 dB and 0.555 dB, respectively.

### 3.2. Side Coupling Structure

**Figure 6:**
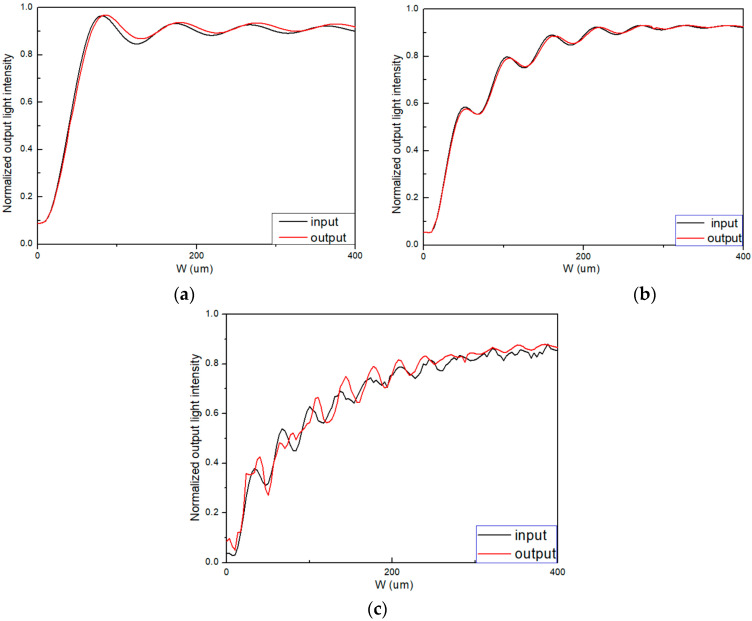
Normalized output and input light intensity with different taper lengths (**a**) input and output taper based on PMMA material; (**b**) input and output taper based on NOA material; (**c**) input and output taper based on SU-8 material.

We used BPM to optimize the length of the input and output tapers to reduce coupling loss. The polymer waveguide in structure 3 employed SU-8, PMMA, and NOA materials. As shown in Figure 6a, for PMMA-based tapers with lengths longer than 200 μm (L_3_ = L_4_ > 200 μm), the coupling loss was 0.286 dB. As shown in Figure 6b, for NOA-based tapers with lengths longer than 250 μm (L_3_ = L_4_ > 250 μm), the coupling loss was 0.310 dB. As shown in Figure 6c, for SU-8-based tapers with lengths longer than 400 μm (L_3_ = L_4_ > 400 μm), the coupling loss was 0.525 dB.

According to the simulation results in Figure 6, we designed three sensors. Structure 3 was utilized for sensor 5, sensor 6, and sensor 7, with PMMA, NOA and SU-8 chosen as the polymer materials for sensor 5, sensor 6, and sensor 7, respectively. To reduce coupling loss in these three sensors, the lengths of their reference and sensing arms were L_1_ = L_2_ = 5000 μm, the widths of the input and output tapers were W_1_ = W_2_ = 3 μm, and the lengths of the input and output tapers for sensor 5, sensor 6, and sensor 7 were 200 μm, 250 μm, and 400 μm, respectively.

**Figure 7:**
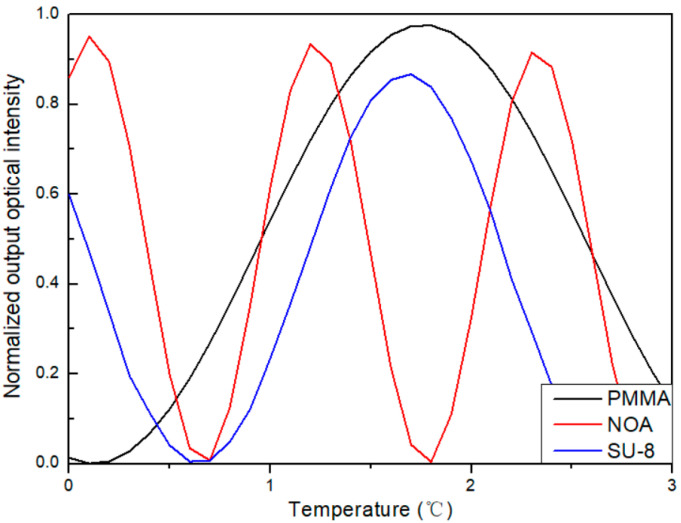
The relationship between the normalized output optical intensity with temperature at wavelengths of 1550 nm.

Next, the temperature sensing performance of the three sensors was analyzed by the BPM. The relationship between normalized output light intensity and temperature at a wavelength of 1550 nm is shown in Figure 7. As shown in Figure 7, the black line represents the simulation data of sensor 5, the red line represents sensor 6, and the blue line represents sensor 7. The temperatures required for achieving π-phase modulation were 1.7 °C, 0.6 °C, and 1.0 °C, with loss of 0.286 dB, 0.310 dB, and 0.525 dB, respectively.

## 4. Discussion

Compared with the direct coupling structure, the side coupling structure exhibits lower loss, higher manufacturing tolerance, and a wider adaptation range of polymer refractive indices. Therefore, this paper further analyzed the sensitivity of the three sensors using the side coupling structure.

(4) $$TP=\lambda_{0}{\left(\frac{\partial n_{eff,1}}{\partial T}L_{1}-\frac{\partial n_{eff,2}}{\partial T}L_{2}\right)}^{-1},$$

(5) $$FSR=\frac{\lambda_{0}^{2}}{n_{g,1}L_{1}-n_{g,2}L_{2}},$$

(6) $$S=\frac{FSR}{TP}=\lambda_{0}\left(\frac{\partial n_{eff,1}}{\partial T}L_{1}-\frac{\partial n_{eff,2}}{\partial T}L_{2}\right){\left(n_{g,1}L_{1}-n_{g,2}L_{2}\right)}^{-1},$$

where $\lambda_{0}$ is the interference wavelength, $L_{1}$ ($L_{2}$) is the length of the reference arm (sensing arm), and $n_{g,1}$ ($n_{g,2}$) and $n_{eff,1}$ ($n_{eff,2}$) correspond to the group index and the effective index of the mode propagating in the reference arm (sensing arm), respectively. When the two arms are of equal lengths, i.e., $L_{1}=L_{2}$, Equation (6) can be written as follows:

(7) $$S=\lambda_{0}\left(\frac{\partial n_{eff,1}}{\partial T}-\frac{\partial n_{eff,2}}{\partial T}\right){\left(n_{g,1}-n_{g,2}\right)}^{-1},$$

According to Equation (7), it can be determined that $\Delta \partial n_{eff}/\partial T$ is positively correlated with S and $\Delta n_{g}$ is negatively correlated with S. We substituted $\partial n_{eff}/\partial T$ and $n_{g}$ data (Table 1) into Equation (7). The sensitivities S of sensor 5, sensor 6, and sensor 7 were found to be −6.85 nm/K, −6.48 nm/K, and −2.30 nm/K, respectively. Compared to devices consisting of a single polymer, this device is an order of magnitude more sensitive. In addition, the group refractive index plays a significant role in S. The closer the group refractive index values of the two material arms, the greater the S value.

The second method of “side-of-fringe mode” temperature measurement [18] is called the temperature responsivity $R_{T}$. We define it as the maximum-intensity response of the device normalized by the maximum path length in the interferometer, such that

(8) $$R_{T}=\frac{\lambda_{0}}{\pi I_{0}\max\left\{L_{1},L_{2}\right\}}\max\left\{\left|\frac{\partial I\left(\lambda \right)}{\partial T}\right|\right\}=\frac{\lambda_{0}}{TP\times \max\left\{L_{1},L_{2}\right\}},$$

where I is the output intensity from a narrow-band input source of intensity $I_{0}$. The wavelength $\lambda_{0}$ is chosen to maximize ∂I/∂T.

When the $n_{g,1}L_{1}=n_{g,2}L_{2}$ ($n_{g,1}>n_{ng,2}$), and thus S, FSR=∞, we can further express

(9) $$R_{T}\left(FSR=\infty \right)=\left|\frac{\partial n_{1}}{\partial T}\frac{n_{g,1}}{n_{g,2}}-\frac{\partial n_{2}}{\partial T}\right|,$$

The sensitivity of the temperature sensor could be significantly improved with a better path length of the interferometer. The limit of temperature responsivity of the three sensors could be calculated by Equation (9). Sensor 5, sensor 6, and sensor 7 were 13.16 × 10^−5^/K, 32.20 × 10^−5^/K, and 20.20 × 10^−5^/K, respectively. The performance of the reported device in terms of temperature responsivity is compared with several previous results using MZIs in Table 2. Our sensor can provide a significant temperature response, thanks to the very large thermo-refractive coefficients between the cores of the silica and polymer waveguides; the larger the difference in thermo-optic coefficients between the two arms of the material, the higher the $R_{T}$ value.

## 5. Conclusions

In this paper, three asymmetric MZI structures composed of a polymer sensing arm and a silica sensing arm were proposed. The loss properties of the direct coupling structure and the side coupling structure were optimized. The results show that the side coupling structure had a lower loss and higher manufacturing tolerance. Sensor 5, sensor 6, and sensor 7 were side coupling structures, utilizing PMMA, NOA, and SU-8 as the polymer materials for each sensor, respectively. The observed losses are 0.286 dB, 0.310 dB, and 0.525 dB, respectively, when the lengths of the reference and sensing arms of sensor 5, sensor 6, and sensor 7 were 5000 μm; the widths of the input and output tapers were 3 μm; and the taper lengths were 200 μm, 250 μm, and 400 μm, respectively.

Comparing the results with previous studies, we found that the sensitivities of asymmetric MZI sensors made of different materials were improved by an order of magnitude compared to asymmetric MZI sensors made of the same material. Sensor 5 based on PMMA materials had the highest sensitivity of −6.85 nm/K due to the close refractive index of PMMA to the doped silica. In addition, the ideal path temperature responsivity (RT(FSR→∞)) of the device was significantly enhanced due to the opposite thermal optical coefficients of polymers and silica. Sensor 6, which was based on NOA materials, achieved an RT of up to 32.20 × 10^−5^/K due to the large thermo-optic coefficient of the NOA material, which suggests that the heterogeneous hybrid integrated MZI structure has a promising application in the field of on-chip temperature sensing.

## Figures and Tables

**Table 1:** Simulated waveguide properties at 1550 nm.

	Silica	PMMA	NOA	SU-8
Dimensions (μm)	3 × 3	3 × 3	3 × 3	3 × 3
$n_{eff}$	1.46	1.47	1.50	1.54
$n_{g}$	1.50	1.52	1.56	1.60
$\frac{dn}{dT}\left(\times 10^{-5}K^{-1}\right)$	1.0	−12	−30	−18
$\frac{dn_{eff}}{dT}\left(\times 10^{-5}1/K\right)$	−2.45	−11.29	−27.56	−17.29

**Table 2:** Comparison of other MZI optical waveguide temperature sensors.

Reference	Method	S (nm/K)	R_T_ (×10^−5^ K)	R_T_ (FSR→∞) (×10^−5^ K)
Sensor 5	PMMA and silica MZI	−6.85	13	13.16
Sensor 6	NOA and silica MZI	−6.48	31	32.20
Sensor 7	SU-8 and silica MZI	−2.30	19	20.20
[19]	Waveguide-width engineered silicon MZI	0.438	0.17	1.0
[8]	Waveguide-width engineered silicon AMZI	0.445	3.7	5.5
[17]	Si MZI with SU-8 cladding	−0.172	-	-
[20]	Si MZI with TiO_2_ cladding	0.34	21.3	-
[6]	NOA MZI	−0.43	-	-

## Data Availability

The original contributions presented in the study are included in the article, further inquiries can be directed to the corresponding author.

## References

[1] Joo J., Park J., Kim G. (2018). Cost-Effective 2 × 2 Silicon Nitride Mach-Zehnder Interferometric (MZI) Thermo-Optic Switch. IEEE Photonics Technol. Lett..

[2] Kim H.-T., Yu M. (2016). Cascaded ring resonator-based temperature sensor with simultaneously enhanced sensitivity and range. Opt. Express.

[3] Benéitez N.T., Missinne J., Shi Y., Chiesura G., Luyckx G., Degrieck J., Steenberge G.V. (2016). Highly Sensitive Waveguide Bragg Grating Temperature Sensor Using Hybrid Polymers. IEEE Photonics Technol. Lett..

[4] Zhu H.H., Yue Y.H., Wang Y.J., Zhang M., Shao L.Y., He J.J., Li M.Y. (2017). High-sensitivity optical sensors based on cascaded reflective MZIs and microring resonators. Opt. Express.

[5] Liu K., Zhang C., Mu S., Wang S., Sorger V.J. (2016). Iwo-dimensionaldesign and analysisof trench-couplerbased Silicon Mach-Zehnder thermo-optic switch. Opt. Express.

[6] Niu D., Wang X., Sun S., Jiang M., Xu Q., Wang E., Wu Y., Zhang D. (2018). Polymer/silica hybrid waveguide temperature sensor based on asymmetric Mach-Zehnder interferometer. J. Opt..

[7] Niu D., Wang L., Xu Q., Jiang M., Wang X., Sun X., Wang F., Zhang D. (2019). Ultra-sensitive polymeric waveguide temperature sensor based on asymmetric Mach–Zehnder interferometer. Appl. Opt..

[8] Ding Z., Shi Y. (2021). Demonstrationof an Ulra-Sensitive Temperature Sensor Using an Asymmetric Mach-ZehnderInterferometer. IEEE Photonics J..

[9] Doerr C.R., Okamoto K. (2006). Advances in Silica Planar Lightwave Circuits. J. Light. Technol..

[10] Cao Y., Zhang D., Yang Y., Lin B., Lv J., Wang E., Yang X., Yi Y. (2020). Au Nanoparticles-Doped Polymer All-Optical Switches Based on Photothermal Effects. Polymers.

[11] Liang L., Zheng C.-I., Sun X.-Q., Wang E., Ma C.-S., Zhang D.-M. (2012). Driving-Noise-Tolerant Broadband Polymer/Silica Hybrid Thermo-Optic Switch withLow Power Consumption. Fiber Integr. Opt..

[12] La T.L., Bui B.N., Nguyen T.T.N., Pham T.L., Tran Q.I., Tong Q.C., Mikulich A., Nguyen T.P., Nguyen T.T.I., Lai N.D. (2023). Designand Realization of Polymeric Waveguide/Microring Structures for Telecommunication Domain. Micromachines.

[13] Xie Y., Han J., Qin T., Ge X., Wu X., Liu L., Wu X., Yi Y. (2022). Low Power Consumption Hybrid-Integrated Thermo-Optic Switch with Polymer Cladding and Silica Waveguide Core. Polymers.

[14] Liu Y., Sun Y., Yi Y. (2017). All polymer asymmetric Mach-Zehnder interferometer waveguide sensor by imprinting bonding and laser polishing. Chin. Phys. B.

[15] Niu D., Zhang D., Wang L., Lian T., Jiang M., Sun X., Li Z., Wang X. (2019). High-resolution and fast-response optical waveguide temperature sensor using asymmetric Mach-Zehnder interferometer structure. Sens. Actuators A Phys..

[16] Ritchie A.W., Cox H.J., Gonabadi H.I., Bull S.J., Badyal J.P.S. (2021). Tunable High Refractive Index Polymer Hybrid and Polymer-Inorganic Nanocomposite Coatings. ACS Appl. Mater. Interfaces.

[17] Guan X., Wang X., Frandsen L.H. (2016). Optical temperature sensor with enhanced sensitivity by employing hybrid waveguides inasilicon Mach-Zehnder interferometer. Opt. Express.

[18] Payne D.A., Matthews J.C.F. (2022). A CMOS-compatible heterogeneous interferometer for chip-scale temperature sensing. Appl. Phys. Lett..

[19] Zhang Y., Zou J., He J.-J. (2018). Temperature sensor with enhanced sensitivity based onsilicon Mach-Zehnder interferometer with waveguide group index engineering. Opt. Express.

[20] Jong-Moo L. (2015). Ultrahigh Temperature-Sensitive Silicon MZI with Titania Cladding. Front. Mater..

